# Home Care in the Daily Lives of Older People: Protocol for an Ethnographic Two-Year Longitudinal Study

**DOI:** 10.2196/42160

**Published:** 2023-03-01

**Authors:** Tove Harnett, Håkan Jönson, Alexander Fäldtman

**Affiliations:** 1 School of Social Work Lund University Lund Sweden

**Keywords:** eldercare, user perspective, home care, theory of care use

## Abstract

**Background:**

Research on eldercare has been dominated by a provider-oriented perspective, concerned with the conditions and views of care providers. There are striking differences compared with the field of disability studies, where help is framed as part of a larger project of having a daily life and being included in society. Pilot interviews indicate that older people develop active strategies to make care work. These include *practical* preparations, *emotional* activities such as showing an interest in staff members’ lives, or *rhetorical* skills in asking for help.

**Objective:**

The aim of this project is to develop empirical and theoretical knowledge of eldercare as a relational practice, accomplished by older people in their daily lives. This perspective will also offer an alternative to ongoing attempts to reduce the user perspective to an issue about older people acting as customers in a market.

**Methods:**

The project will map, investigate, and follow up on care use from the perspective of care users. The project has an ethnographic 2-year longitudinal approach. Data consist of interviews and participant observations with 35 persons (home care users and cohabitating partners) and a diary study with additional 10 care users. Inclusion criteria are people 65 years or older with home care provided by needs assessors. It is preferred that they have had home care between 6 months and 2 years in order to follow a progression in roles, identities, and strategies within home care use.

**Results:**

Between May and October 2022, 25 interviews with home care users were conducted. Data collection with follow-up interviews and observations, analysis, and reporting of findings will be completed by December 2024.

**Conclusions:**

By studying care use in the context of older people’s lives the project will add important knowledge about the strategies and adjustments older people apply to make care arrangements work. A user-oriented perspective will further the understanding of how power relations play out in home care over time in relation to the formal rights, categorical belongings, and established norm systems that place the user in superior or subordinate positions.

**International Registered Report Identifier (IRRID):**

PRR1-10.2196/42160

## Introduction

### Background

Care has been studied as a relational practice, but above all as a practice for those who *provide* care. This project will shift focus to go beyond the casting of older people as either passive objects or active consumers to provide the empirical and theoretical base for a third route. The point of departure is that older persons act and reason in specific ways to make home care work for them and to ensure that help is provided without friction. This entails thoughts, deliberations, and a range of actions that for older people with home care are part of everyday life:

- Waking up early to prepare the home for visiting care staff
- Learning the home care organizations’ technical vocabulary
- Working out which care staff will take out the rubbish as they leave, even if this service is not officially included
- Showing an interest in the personal lives of the care staff and finding common interests
- Deciding whether to go grocery shopping in person or to adapt to buying food online
- Deciding to buy in tax-deductible cleaning and laundry services privately to supplement council-provided home care. (Tax deduction on household services or RUT (rengöring, underhåll, och tvätt [cleaning, maintenance, and washing] were introduced in Sweden in 2007, and RUT service companies increasingly use the term “eldercare” to promote their services.)

Research on home care and other forms of eldercare has been dominated by a provider-oriented perspective, which revolves around the organization, conditions, views, and activities of those who provide the care. This perspective has been developed in theories that define care as a moral, relational practice by which the caregiver interprets the needs of the dependent person [1-6]. Studies of the everyday realities of home care have comprised both providers and users, but as in the excellent thesis by Szebehely [7], the analytical focus and the theorization of eldercare have usually been directed toward the provider side of the relationship. This perspective has also dominated studies of the coordination of care efforts, informal care, and its nexus of formal care [8,9]. Even approaches in the “person-centered” care paradigm share this focus; one review found that most models measured staff members’ views [10]. Although the provider-oriented perspective has enlarged our knowledge of the everyday realities of care, few studies have considered the care users’ perspectives. The absence of a user-oriented perspective is significant when compared with the field of disability studies. In a review of Swedish research, Erlandsson [11] noted that of 26 Swedish theses about “non-old” people with disabilities, the sole focus of 11 was the person being helped and 2 focused on the provider. By contrast, of the 25 Swedish theses about care for older people, none focused only on the user, whereas the sole focus of 12 was the care provider. The provider-oriented perspective in eldercare is strikingly different from approaches in disability studies. One reason for this is that help for “non-old” people is framed as part of a larger project of daily life and being included in society [11-13]. The provider perspective is crucial in any care situation, but too strong an emphasis on providers plainly risks discounting aspects such as social rights and undermining attempts to resist ageism and exclusion.

This project will address the need for a user-oriented perspective as part of a larger mission to develop a theory of care use. A user-oriented perspective will further the understanding of how power relations play out in home care over time, in relation to the formal rights, categorical belongings, and established norm systems that place the user in superior or subordinate positions. The perspective will also offer an alternative to ongoing attempts to make sense of the meaning of eldercare in a new landscape, where new cohorts of older people become care users, and where marketization has been presented as both a solution and a threat to the welfare state’s ambitions [14-16]. Critical approaches have provided valuable knowledge, but in the attempt to combat unrealistic forms of marketization [17], there is an evident risk of downplaying the capabilities of older care users. An alternative route is needed that acknowledges the importance of relations, skills, and adjustments over time.

The project will use qualitative interviews, participant observations, and a diary study to investigate understanding, strategies, and adjustments of older people who use home care, thereby providing a type of knowledge that has so far not been devoted sufficient attention.

### Aim and Research Questions

The aim of the project is to develop empirical and theoretical knowledge of home care as a relational practice, as interpreted, accomplished, and negotiated by older people in their daily lives, as part of the formal and informal services available to them:

- RQ1: How do older people navigate, coordinate, and fit care arrangements and other forms of help into their home contexts, daily activities, long-term plans, and social relations?
- RQ2: What practical, emotional, and rhetorical strategies do older people apply in their interaction with care staff?
- RQ3: What types of identities, categorizations, and roles are played out when older people receive home care, and what is the bearing on power relations?
- RQ4: How are users’ needs formulated as part of their experience of the eldercare system and their relations with home care staff over time?

### The Context of Studies

The studies of the project are situated within several fields of research and investigations, internationally and in the Nordic countries.

A comprehensive body of research theorizes and investigates eldercare, with a strong tradition and prominent research environments in Sweden, Denmark, Finland, and Norway [3,5,18-20]. The research has concentrated on the provision of formal eldercare as part of the social policy of the Nordic welfare states, with studies of the everyday realities, organization, working conditions, needs assessments, and relations between formal and informal care. Research and theories that use this provider-oriented perspective will serve as a backdrop to the project.

A second type of research that informs the project consists of studies that gauge care users’ opinions when defining quality of care. The National Board of Health and Welfare’s large annual user survey “Vad tycker äldre om äldreomsorgen?” (What do older people think about eldercare?) is the most prominent study from a Swedish horizon [21]. It should be noted that such studies have been criticized for the way they translate quality into numbers. Internationally, qualitative and mixed methods have been used to investigate how the quality of care is measured or the broader issue of quality of life from the perspective of the care user [22].

A third type of research that will feed into the project consists of studies of user participation, originally developed to empower older people who use health care or social care. This research tradition is anchored in Arnstein’s [23] paradigmatic article on the ladder of participation and includes studies of decision-making, power sharing, and the influence of formal interest groups [24]. Notably, however, that participation is usually constructed in eldercare as involvement in care (such as helping get dressed, and sometimes with the aim to achieve help to self-help), whereas the disability field focuses on participation in society and how society’s support makes that possible [11].

For the project, the state-of-the-art research builds on what Lloyd [25] calls the personalization agenda, also described as a relational-political interpretation of the capability approach [26]. Acknowledging the relevance of structural conditions and care policies, this research has included studies of power and control over services, relations, time, and the home and its artifacts, and shows that home care can be both enabling and restrictive [27,28]. The approach has also included studies with a phenomenological approach that relate the experience of receiving home care to the meaning to older individuals of home, body, dependence, and relations [29]. Several studies have focused specifically on how older people view, evaluate, and work to establish such relations [30]. Studies using this approach have also addressed the shift to marketization and show that older care users’ choices play out in ongoing care and not just at the point when a particular care provider is chosen [31-33].

Drawing on theories in disability studies, the knowledge gained from the project will be used to develop a theory of care. The fact that the nexus of disability and ageing is attracting greater interest, and that concepts such as social rights, discrimination, and exclusion are now being used in policies on ageing, will facilitate the use of a disability lens in the study of home care [11,12,34], bringing a fresh focus on the way home care services enable older people to fully participate in society, or, where relevant, to regard services as restrictive and therefore ageist, with all that the concept of ageism means for the project findings.

The project will take a longitudinal approach. Increased needs frequently entail help from ever-larger numbers of providers, and pilot interviews reveal the efforts made to navigate what is on offer from different providers. Previous studies show that care users adapt their habits to (restrictive) arrangements that they consider to be the conditions of care, but that they also learn what demands and questions to put to care providers, and they try to develop relations that work against and between what is formally granted by the organization [27,28]. The project will provide a unique understanding of how relations, loyalties, skills, and adaptations develop within home care.

## Methods

### Overview

To address its stated aim and questions, the project will map, investigate, and follow up on care use from the perspective of care users. The mapping will be done with structured interviews with users and in some instances with information from their cohabiting partners and is inspired by existing network studies [35]. The main part of the project will be based on interviews and participant observations. Finally, care use will be followed up using the project’s chosen longitudinal approach [36]. This phase of the project will also include a study of care users’ diary notes. In its qualitative and longitudinal approach, the project will provide knowledge beyond satisfaction ratings and so will contribute to a well-grounded understanding of eldercare as negotiated by older people in their daily lives, with developing relations and adaptations. By including cohabiting partners, knowledge will be gained about how they too fit care arrangements into their daily lives, and possibly must take on new roles when interacting with home care staff.

Care always occurs within a broader social context of established policies, here in the context of the Nordic welfare state. Policy is realized in everyday practices, and thus a microsociological understanding is crucial when theorizing care use. The project will be theoretically anchored in symbolic interactionism and will use an ethnographic study design to reveal meaning-making, interaction, and the negotiation of identities. The point of symbolic interactionism is that people act toward things based on the meaning those things have for them, and meaning is created in an interpretive process derived from social interaction [37]. In the data analysis, we will make use of theories and concepts developed for the understanding of human service work [38,39] and care work [1,40]. These theories and concepts will be adapted to the care users’ positions and perspectives, and the analysis will focus on categorization work (users’ categorizations and their evaluations of care staff), empathy, and emotion work, whereas impression management will be studied at the point when older people encounter human service organizations and their representatives (the care staff). This redirecting of analytical focus will be facilitated by the types of comparison to the field of disability studies suggested above.

The theories that the project is designed to develop will increase the understanding of home care use as a *position* and *practice*, anchored in a broader social context but realized in older people’s everyday lives. This way of theorizing care could potentially be developed as part of a broader paradigm of help-receiving and care-receiving that reflects people’s everyday conditions.

Studies that use a symbolic interactionist approach typically have 2 overarching stages: exploration and investigation [37]. Below, we will first describe how the exploration stage has proceeded, to be followed by the investigation stage, which falls into 3 phases. During the investigation, concepts will become fixed as part of the findings and theory development.

### Exploration

The exploration stage has been initiated and conducted through pilot interviews and various contacts with partners for collaboration in the field. The pilot interviews (financed by Helsingborg Council for December 2020 to October 2021) indicate that care users engage in a continuous form of “work”—emotional, practical, and rhetorical activities. Care users use these activities to prepare staff and guide the provision of care, to defend the privacy of their relations and homes, and to avoid depersonalization. Some users carefully prepare their homes and themselves to ensure that care activities run smoothly. They may display an emotional interest in staff members’ personal lives to make themselves visible as individuals. Some users describe rhetorical strategies to adopt the correct organizational and technical vocabulary to get the help they want, to avoid the response “We don’t do that,” and to develop relations with staff that affect the character of care.

From the exploration stage’s pilot interviews and literature review, we have so far identified four themes to be used as sensitizing concepts.

1. Enablement (goals in everyday life, achieved with home care and the navigation and coordination of services)
2. Relations and loyalties (in and beyond the home care tasks)
3. Power and control (over services, space, time, and categorizations)
4. Identity (as affected by receiving care and used to interpret the meaning of care)

The investigation will fall into 3 phases, including empirical studies, collaboration with users and other actors outside academia, and dissemination. The studies of the project are described below, whereas the other activities are described elsewhere in this application.

### Stakeholder Involvement During the Exploration Stage

Contacts with members and stakeholders of the field studied are an essential part of the exploration stage, and in the project, such contacts take the form of user involvement and collaboration with actors outside academia. The project uses a model for collaboration and user involvement that focuses on purpose, actor, magnitude, and process [41]. This PAMP (Purpose, Actor, Magnitude, and Process) model (SAPO in Swedish) will guide the development of the project, and below we describe the way the initial stage has proceeded. The purpose of this involvement is to identify relevant questions and sensitizing concepts for further studies, a process similar to what Greenhalgh et al [42] call priority-setting.

Two stakeholder panels have been set up, and additional collaboration has been established with a panel of stakeholder organizations.

#### Panel 1: Stakeholders With Home Care and Cohabitating Partners

Throughout the project, a user panel of 5 older people with several years of experience of home care will provide feedback on the research process, analysis, and dissemination of results. The panel members have already been involved in the exploration stage, developing research questions and provided valuable feedback on how to strengthen the project’s user orientation. Members of the user panel will be part of the project for the entire period (except the dissemination phase) and will be able to influence findings by their validation at regular meetings. The panel consists of persons from the pilot interviews, the current ethnographic study, and professional and personal contacts. Participants have been informed that participation in the panel is voluntary and based on interest to discuss the type of questions that the project investigates. The plan is to have 2 annual meetings. A first meeting was held in May 2022 and a second in November 2022. A lesson so far concerns the frailty of the panel, with a need to recruit new members in case of severe illness or death.

#### Panel 2: Senior Stakeholders

Throughout the project, a panel of 6 older persons who are interested in the topic of the project will provide feedback on the research process, analysis, and dissemination of results. They will be recruited among senior citizen organizations as well as professional contacts in order to get a balanced distribution of senior citizens. Participants have been informed that participation in the panel is voluntary and based on interest to discuss the type of questions that the project investigates. The plan is to have 2 annual meetings, and the meetings were held in April 2022 and October 2022.

#### Panel 3: Organizations as Stakeholders

Contact has been established with 3 collaboration partners to act as members of a reference group during the projects. These partners are representatives of Helsingborg Council’s eldercare provision; regional and local representatives of the 2 pensioners’ organizations, PRO (Pensionärernas riksorganisation, the Swedish national pensioners’ organization) and SPF (SPF-Seniorerna, the Swedish association for senior citizens); and representatives of Seniorval (lit. Seniors’ choice), a national private provider of information and guidance for older people who apply for home care. During the investigation and dissemination stage, the external partners will be invited for discussions on how to translate our findings into information and programs that are useful for older people who use home care.

The purpose of involving representatives of a local authority and a provider of information—Seniorval—is to achieve direct impact by developing routines and information based on the findings of the project. These actors will be consulted at regular meetings.

### Investigation

#### Phase 1: Mapping Care Arrangements and Investigating Care as a Relational Practice

The first core study will map out the different kinds of care arrangements in older people’s everyday lives. Research on informal care has shown that relatives provide a type of managerial care that includes the planning and coordination of help efforts [9], and it has been noted that also older home care users themselves and their cohabiting partners engage in such coordinating efforts [28].

Study 1 will be based on semistructured, qualitative interviews with 25 care users and 10 cohabiting partners, in which the research questions will serve as nodal points for the questions in the interview guide. The first part of each interview will be devoted to a structured mapping of help from the perspective of the care user (including cohabiting partners’ perspectives on the help provided in their shared home).

#### Phase 2: Home Care as Accomplished, Coordinated, and Fitted Into Everyday Life

The second core study will be the most extensive, to be based on interviews and participant observations on several occasions. Researchers will be present in the users’ homes on 2 separate occasions when care staff visit. During these “stay-along observations” (inspired by go-alongs [43]; see also [28]) we will observe and engage in informal field-based interviews with users, staff, and (where present) cohabitating partners. This method will allow us to observe users’ preparations and interactions, matched with a fine granularity in the details of care for the necessary contextual understanding of the users’ and cohabiting partners’ interpretations and strategies.

Study 2 will be based on the interviews conducted in study 1 and on 2 participant observations in the homes of the 25 care users within a month of the first interviews. Instead of accompanying home care staff as they visit various users, we will be present in the users’ homes on 2 separate occasions when home care staff visit.

#### Phase 3: Care Use and Change Over Time

The third stage of the project will follow up on care use using the project’s chosen longitudinal approach [36]. On the basis of interviews with care users and additional observations, this approach will provide knowledge of how the user’s strategies, perceived needs, and relationships with care staff change. A second study with the same objective will collect data in the form of older care users’ diaries. Diary methods can offer a more comprehensive understanding of an individual’s everyday activities and thoughts, and the data provided are likely to give a different insight compared with conventional interview approaches [44]. It is also a means of identifying important but seemingly mundane practices, which may be overlooked when relying on “snapshot” approaches such as interviews and surveys.

The follow-up study is based on interviews with all available care users, conducted 16-22 months after study 1. The objective is to gauge how perceptions, relations, and strategies change over time, especially in relation to any alteration in the users’ needs and degree of dependence. A total of 10 participants will be chosen for a final round of participant observations.

Diary data will be collected from approximately 10 care users. Each participant will be asked to complete and return a weekly diary for 20 weeks.

### Data Collection

Approximately 25 care users from three Swedish local authorities will be included in the project. Additional interviews will be conducted with 10 cohabiting partners. Out of the 35 total respondents, data will thus consist of 60 (25 + 25 + 10) interviews plus 60 (25 + 25 + 10) stay-along observations and diary notes from 10 home care users. The following selection criteria will be used to identify participants: the care user should be 65 years or older and have home care at least twice a week. The project will strive to achieve diversity in the level of care, gender, socioeconomic aspects, and length in experience of living with home care. As part of the effort to map services from a user perspective, 10 participants will be chosen who have home care and combine it with private help through the RUT.

Approximately 10 older people will participate by writing diary notes about their experiences of home care and other forms of care over 20 weeks. The following selection criteria will be used: the care user should be 65 years or older, have home care at least twice a week, and be interested in formulating their experiences in writing. Participants will be recruited through the website Seniorval, where the researchers will advertise for home care users with an interest in documenting their experiences and reflections in diary format.

Home care users with cognitive disorders (dementia) will not be included in the project studies, but the project will develop theory that may be used in future studies of persons with such disorders.

The types of data that are generated are recorded and transcribed interviews, field notes from observations, and diary entries.

### Data Analysis

The project will produce a range of findings and is designed to develop a theory of care use. Data will be coded using the NVivo software package, and the steps of qualitative content analysis [45] will be used in the different project studies. Following the tradition of symbolic interactionism, findings will be grounded in the data, guided by the research questions of the project and by the sensitizing concepts identified during the exploration stage. Theories of care work and categorizations that relate to the tradition of symbolic interactionism will inspire the analysis and development of the theory. See also the section on theoretical foundations and guiding tools above.

### Ethics Approval

The project plan has been approved by the Swedish authority of ethics approval (2022-00829-02).

The project will focus on how older people interpret and negotiate home care in their daily lives. Thus, it will likely generate sensitive personal data on health. This will be the case, for instance, if interviewees comment and elaborate on illness, disability, dependency, and well-being. The principal investigator and co–principal investigator are familiar with the content and procedures for such applications and the proper procedures that follow ethical approval. Research participants will be provided with written and verbal information about the project’s studies, they will be informed that participation is voluntary, and they will be asked to sign a consent form. Data will be anonymized, and publications will not contain information that makes it possible to identify individuals.

The members of the research team have extensive experience in interviewing older people and observations in sensitive contexts. Our experience is that project information and informed consent should be treated as continual practices throughout the research process. Given the project’s longitudinal approach, researchers will ask for informed consent at each of the 4 visits. Prior to each “stay-along observation,” the researcher will ask research subjects about potentially sensitive situations and how these should be handled. Researchers will not observe situations that are ethically problematic, such as toilet visits and dressing or undressing.

The knowledge gained will benefit older people in a broad sense by shedding light on the everyday realities of those who receive home care. Hitherto, the absence of a true user perspective is striking. The project is not expected to cause any harm or inconvenience to the research subjects but will hopefully lead to improvements in the long run for older people with home care.

## Results

Members of panel 2 participated in a meeting with representatives of the project in April 2022, with the aim to introduce the project. A meeting with panel 1 was held in May 2022, where members of the group provided feedback on an interview guide, through meta-discussions and direct responses to some of the questions. A second meeting with the 2 panels was held in October and November 2022, with the aim to present preliminary findings in order to discuss their validity and relevance.

Between May and October 2022, the first round of interviews (n=25) was finalized. Interviews with partners will be finalized in January 2023, and the first round of observation is expected to be finalized by March 2023. Data collection with follow-up interviews and observations, analysis, and reporting of findings will be completed by December 2024.

## Discussion

### Principal Findings

The expected main findings of this ethnographic study on home care from the user perspective are that older people use their agency to navigate and fit home care into their daily lives, and that home care enables aging in place but has a number of restricting features that concern the control of time and place.

A user-oriented perspective on eldercare will have an important role in the new landscape of eldercare. This landscape has a theoretical and administrative ambiguity about the content and meaning of eldercare. A revision of the Social Services Act [46] in 2018 allows local authorities to grant older persons home care without an assessment of individual needs. This reform is likely to push the assessment of needs toward the actual meeting between the care user and the care provider, a development at odds with the emphasis on professional needs assessments according to the International Classification of Functioning, Disability and Health–based tool IBIC (Individens behov i centrum [the Individual’s needs in center]), previously introduced by Socialstyrelsen [47]. The emphasis on the actual meeting or interaction between the user and provider is also evident in the government white paper [48], according to which older home care users should be offered a personal contact person (*omsorgskontakt*) who is responsible for providing support and managing the personalization of care. However, the actual nature of the position of *omsorgskontakt* is left open. Should it be a member of the professional team that coordinates services for the user, or should it be a care worker who is chosen by the user to perform most services based on their relation and trust? This role ambiguity is also connected to the ongoing marketization of eldercare. Should care be provided by certified, professional care staff, as has been suggested in a government investigation [49] or should Sweden’s system for tax deductions for domestic services (RUT), which is increasingly being used and sometimes labeled as “eldercare,” be further developed for the category of older people [50]? This system has made it cheaper for older people with higher incomes to buy help and personal care or to top up needs-assessed home care [15]. This development may cause increased social inequality and the exploitation of unskilled workers—private care is now the backbone of eldercare in several European countries. Within the heated debate on marketization, there is even a risk that researchers stress the inability and incompetence of older people as care users to question the relevance of choice models [17]. The third route that the project will develop is needed as part of the ongoing development of eldercare.

### Strengths and Limitations

The project has a qualitative multimethod approach using semistructured interviews, observations, and diary notes. A strength in method diversification is a prediction of each capturing different aspects of the subjective experience. An interview is more likely to prime a respondent to evoke stories of critical or otherwise noteworthy events, mostly because of role expectation. The collection of diary notes aims to capture more mundane observations from an older person that may not have made it into an interview answer. The added strength of a period of diary note taking is that the older person will likely be more observant for a period of time. The observations complement the other methods, in that the researchers observe what is happening and prompt a series of short field-based interviews [51] right after a home care visit, which is expected to give slightly different answers when the memory and possibly affect are fresh. The mapping aspect has the strength of combining older persons’ experiences with more structured questions from the researcher, contrasting the older persons’ subjective experience with references of comparative needs [52].

The main limitation of the project concerns recruitment and inclusion criteria. For the selection of the first 35 older persons for interviewing, the municipal home care managers are needed in order to gain access according to plan. By having municipal managers and home care staff as gatekeepers, there is a risk that older persons who are most likely to have a positive attitude toward home care will be asked to participate. This risk is particularly present when organizations represent fear that the project aims to evaluate the organization and its co-workers rather than exploring the experience of everyday life with home care. Representatives will therefore be informed about the aims of the project and that data will be anonymized when presented in publications. Alternative forms of recruitment based on advertising and snowball sampling will be used as complement, but it should be acknowledged that these methods are likely to result in an overrepresentation of persons being particularly critical. Finally, persons living with dementia and mental illness are excluded from the project for ethical reasons, unless they appear in interviews with cohabiting partners. This excludes an important part of the population who uses home care. These problems of representations will be continuously discussed among members of the research team and presented as limitations in publications of the project.

### Conclusions

In this ethnographic 2-year study on home care from a user perspective, we will interview older persons on 2 occasions, conduct participant observations and ask care users to write home care diaries.

By studying care use in the context of older people’s lives the project will add important knowledge on the strategies and adjustments older people use to make care arrangements work. A user-oriented perspective will further the understanding of how power relations play out in home care over time, in relation to the formal rights, categorical belongings, and established norm systems that place the user in superior or subordinate positions. The project is expected to contribute empirical and theoretical knowledge on care and is anticipated to have a societal impact on 3 different levels.

First, the quotes and discussions that will be cocreated by respondents and researchers can serve as important consultative data for policy makers. The unique value from a policy design perspective is that the research goes beyond a user-satisfaction focus and gives an older person perspective on how the current welfare delivery is received, interpreted, and negotiated.

The second dimension of contribution is that this research can be read by providers of home care. It may act as consultative data in terms of how the scheduling, staffing patterns, and “the doing” are perceived and how the elderly use their agency to make it fit their life plans. More importantly, it can be used by care staff and other providers of care in order to develop a deeper understanding of everyday practices from the perspective of those receiving help.

The third dimension of the societal impact is an emancipatory one, given that older people who use home care can take part in other persons’ stories in order to make sense of their own experiences as well as finding legitimacy, comfort, and courage in knowing that others share aspects of their subjective experiences. A long-term secondary outcome may be a contribution toward mobilization and gaining leverage in maneuvering a quasi-market of welfare provision.

## Appendix

Multimedia Appendix 1 Peer review report by Forskningsrådet för hälsa, arbetsliv och välfärd / Swedish Research Council for Health, Working Life and Welfare (FORTE).

## Abbreviations

RUT: Rengöring, underhåll och tvätt (cleaning, maintenance, and washing)

## Data Availability

All data are qualitative and no data sets have been used. According to the project plan’s ethics approval, the transcribed interviews are available to the responsible research team.
