# Supplementary material for: Home Care in the Daily Lives of Older People: Protocol for an Ethnographic Two-Year Longitudinal Study
Source: JMIR Res Protoc. 2023 Mar 1;12:e42160. doi: 10.2196/42160 (PMC10018379; doi:10.2196/42160)
Supplement: Multimedia Appendix 1 [file resprot_v12i1e42160_app1.pdf]

FORTE:
Forskningsrådet för hälsa, arbetsliv och välfärd

Beredningsgruppens yttrande

2021-00506	Tove Harnett	Beredningsgrupp: SOA 2021

Utlysningsnamn: Forskningsprojekt 2021

Bidragsform: Projekt

Projekttitel (svenska): Hemtjänst i äldres vardag: Ett brukarorienterat perspektiv

Sökt inriktning: Fritt

Bedömning

Syfte, frågeställningar, teorianknytning, bakgrund och projektets originalitet

This is a well written application and the project seems solid. The aim of the project is to develop empirical and theoretical knowledge of care as a relational practice, interpreted, accomplished, and negotiated by older people in their daily lives. In doing so, the applicants aim to add a user perspective to the study of care use, as is more often found in disability research. The application includes a number of highly relevant research questions and is based on an appropriate theoretical frame. The study could benefit from a focus on the policy/practice context and how care use and care relations are often fragmented and discontinued, for instance with lack of continuity in staff and limited time for visits. Also a gender focus would have been appropriate.

Studiedesign, metoder för datainsamling och analys

The study is based on interviews and observations with 25 study participants 65+ in addition to diaries in the last phase of the study. The applicants argue that the study is longitudinal but thus apply different methods of data collection across the study, why data will not be fully comparable. There is some weakness in the description of data collection and analysis.

Köns- och genusperspektiv i forskningens innehåll

This is considered in the study but not part of the approach, for instance whether there is a gender dimension in home care use as a position and practice.

Genomförbarhet

The study seems feasible although substantial parts of the project will be conducted by an unknown person.

Relevans, samverkan och nyttiggörande

The study is relevant in studying home care use from the users perspective and with a number of analytical and theoretical focus points which are more often applied in disability research. The project involves a user panel of home care users 75+ who will be part of planning the research design. It is not explained why the user panel is older in age than the participants. There willl also be a reference group with pensioner organisations and provider organisations. 5 articles will be produced.

Sammanfattande bedömning

Overall, the study is ambitious and highly relevant and with some but minor flaws. The proposal is recommended for funding.

Förslag till beslut (bevilja, reserv, avslå)

Approve
